# Effects of FSH and the weather during induced ovulation and timed artificial insemination to increase jenny conception rates

**DOI:** 10.1038/s41598-019-39757-3

**Published:** 2019-03-01

**Authors:** Zhong-Le Chang, Bao-Xing Li, Bing Liu, Luo Yao, Jie Yu, G. M. Jiang, Jing-He Tan

**Affiliations:** 1 Shandong Provincial Key Laboratory of Animal Biotechnology and Disease Control and Prevention, College of Animal Science and Veterinary Medicine, Shandong Agricultural University, Tai’an City, 271018 P. R. China; 2 Dong e e Jiao Co., Ltd., Dong’e County, 252200 Shandong Province, P. R. China

## Abstract

Experiments were conducted to improve jenny conception rates through induced ovulation and timed insemination. Jennies in control, hCG and LH groups were injected intramuscularly with saline alone or saline containing hCG or LH, respectively, when the dominant follicle diameter reached 35 mm. Then, follicle development was checked every 8 h until the dominant follicle ovulated. While 76% of the hCG-treated jennies ovulated between 24 and 48 h, and 84% of the LH-treated ovulated between 24 and 40 h after injection, ovulations in control jennies scattered over an extended period after injection. Conception rates after insemination were significantly higher in LH- or hCG-treated jennies than in the conventionally-bred jennies. The LH preparation used in this study contained more FSH than the hCG preparation did, and supplementing the hCG treatment with FSH significantly improved ovulation synchronization. Ovulations in jennies treated on rainy days were significantly postponed and less synchronized compared to those in jennies treated on sunny days. Together, the results suggested that jenny conception could be significantly improved by inducing ovulation with LH or hCG treatment followed by timed insemination and that FSH and the weather during treatment had profound effects on ovulation induction of jennies.

## Introduction

Breeding management is of great importance for the development of the donkey industry. However, the breeding management in donkey is still a challenge due to limited research involving the jenny. Although artificial insemination (AI) in equids was carried out in early studies, it was not as successful as that conducted in other domestic species. Thus, induction of ovulation in a short predictable time is of great importance for enhancing the reproduction efficiency of equids including the donkey. Furthermore, the precise mechanisms that induce follicular maturation and ovulation in equids are largely unknown.

Several hormones have been tested to induce ovulation in equids, including human chorionic gonadotropin (hCG), crude equine gonadotropin (CEG) and gonadotropin-releasing hormone (GnRH). Among these hormones, hCG is the most commonly used^1–3^ that produced satisfactory results^4,5^. However, some studies suggested that repeated administration of hCG could stimulate antibody production and thus decrease its efficacy in the subsequent treatments^1,6^. Therefore, alternative regimes for ovulation induction in equids have been examined. Although CEG has been tried and found consistently inducing ovulation with no side effects^7^, it is not commercially available. The use of GnRH for ovulation induction in mares has been reported with inconsistent results. For example, although Irvine *et al*.^8^ reported that ovulation induction with GnRH was successful in mares, their results were not confirmed by other studies^7,9,10^. Furthermore, conception rates have seldom been observed in jennies or mares following ovulation induction treatment^11^. In addition, whether the interval between hormone administration and ovulation is influenced by the weather during ovulation induction treatment has not been observed.

It is known that LH has a shorter half-life than hCG does^12^, and thus, it may present a milder stimulus than hCG for antibody development. The objectives of this study were therefore (a) to test whether LH could be used to replace hCG for ovulation induction in jennies; (b) to observe conception rates following ovulation induction treatment of jennies; and (c) to determine the effects of the weather during the ovulation induction treatment on ovulation synchronization of jennies.

## Results

### Distribution of ovulations after different treatments of jennies for ovulation induction

**Figure 1:**
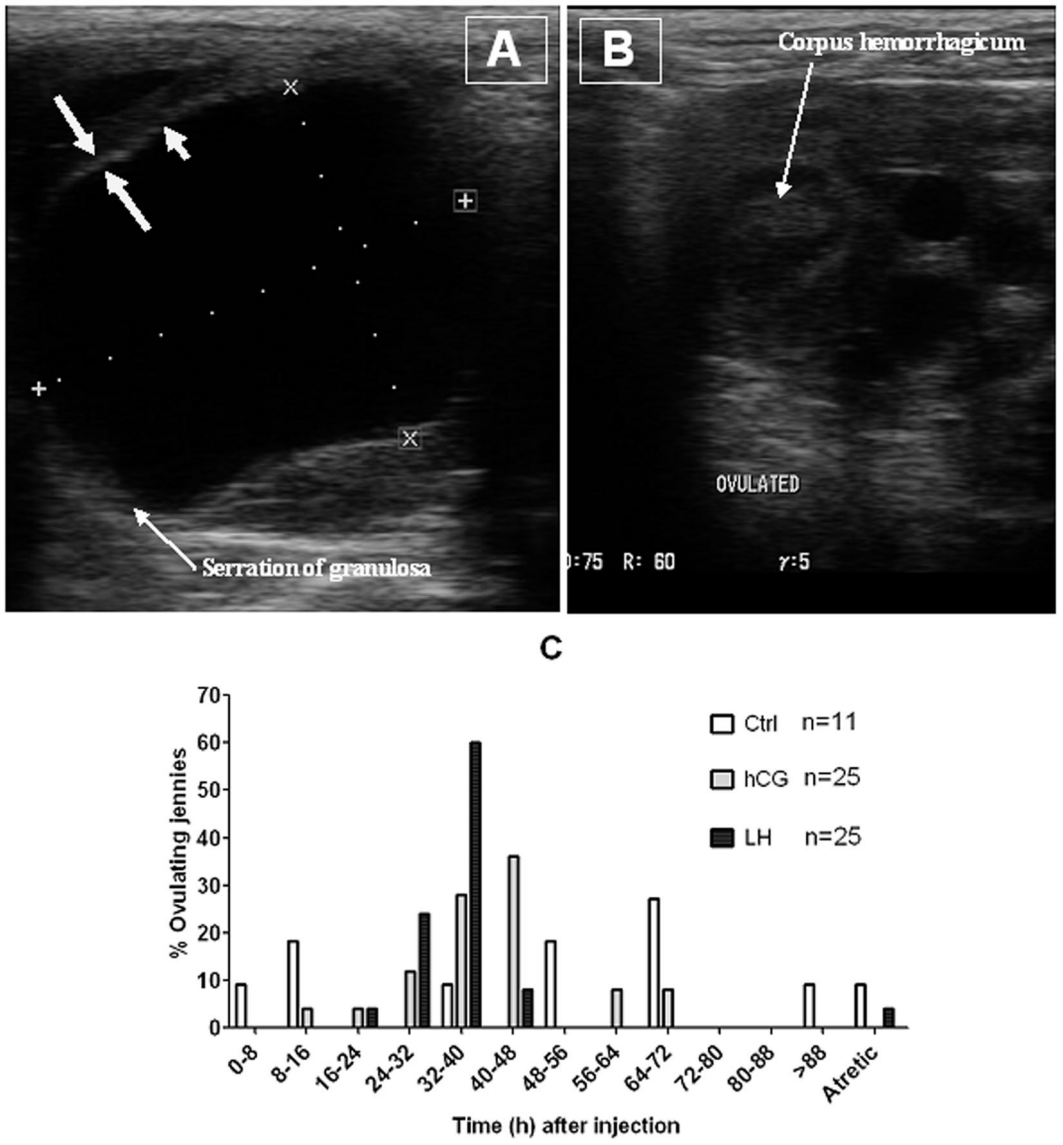
Distribution of ovulations following the ovulation induction treatment of jennies. When the dominant follicle diameter on an ovary reached 35 mm, jennies in control (Ctrl, n = 11), hCG (n = 25) and LH (n = 25) groups were injected intramuscularly with 5 ml saline alone or saline containing 3000 IU hCG or 400 IU LH, respectively. Then, follicle development was checked by ultrasonic imaging every 8 h until the dominant follicle ovulated. (**A**) Ultrasonographic image of a large preovulatory follicle with a thick echogenic border. As ovulation approached, the granulosa layer (large arrows) became thicker (more echogenic), and segments of the follicular wall with an anechoic bands (short arrows) appeared. (**B**) Ultrasonographic image of a collapsed ovulated follicle forming a corpus hemorrhagicum after LH treatment. (**C**) Distribution of ovulations following different ovulation induction treatments.

To observe the effects of different treatments on ovulation synchronization, jennies were checked for follicle development once a day by ultrasonic imaging. When the dominant follicle diameter reached 35 mm, jennies in control, hCG and LH groups were injected intramuscularly with 5 ml saline alone or saline containing 3000 IU hCG or 400 IU LH, respectively. Then, follicle development was checked every 8 h until the dominant follicle ovulated. Ultrasonographic images of a preovulatory follicle before ovulation and an ovulated follicle after LH-induced ovulation are shown in Fig. 1A,B, respectively. Our observations (Fig. 1C) showed that in the control group, 27.3% of the jennies ovulated from 64 to 72 h, and 18.2% ovulated from 8 to 16 h or 48 to 56 h after injection. In the hCG group, 36% of the jennies ovulated from 40 to 48 h, 28% ovulated from 32 to 40 h, and 12% ovulated from 24 to 32 h after injection. In the LH group, 60% of the jennies ovulated from 32 to 40 hours, 24% ovulated from 24 to 32 h, and 8% ovulated from 40 to 48 h after injection. Thus, 76% of the jennies in the hCG group ovulated between 24 and 48 h, and 84% in the LH group ovulated between 24 and 40 h after injection, whereas ovulations in the control jennies were much less concentrated scattering throughout the whole period after injection. Furthermore, our calculation of ovulation windows taking all the ovulations into account indicated that ovulations occurred within 88-h, 64-h and 32-h windows in the control, hCG and LH groups, respectively, confirming further that ovulation was better synchronized with LH than with hCG than in untreated controls.

### Conception rates following different treatments of jennies for ovulation induction

**Figure 2:**
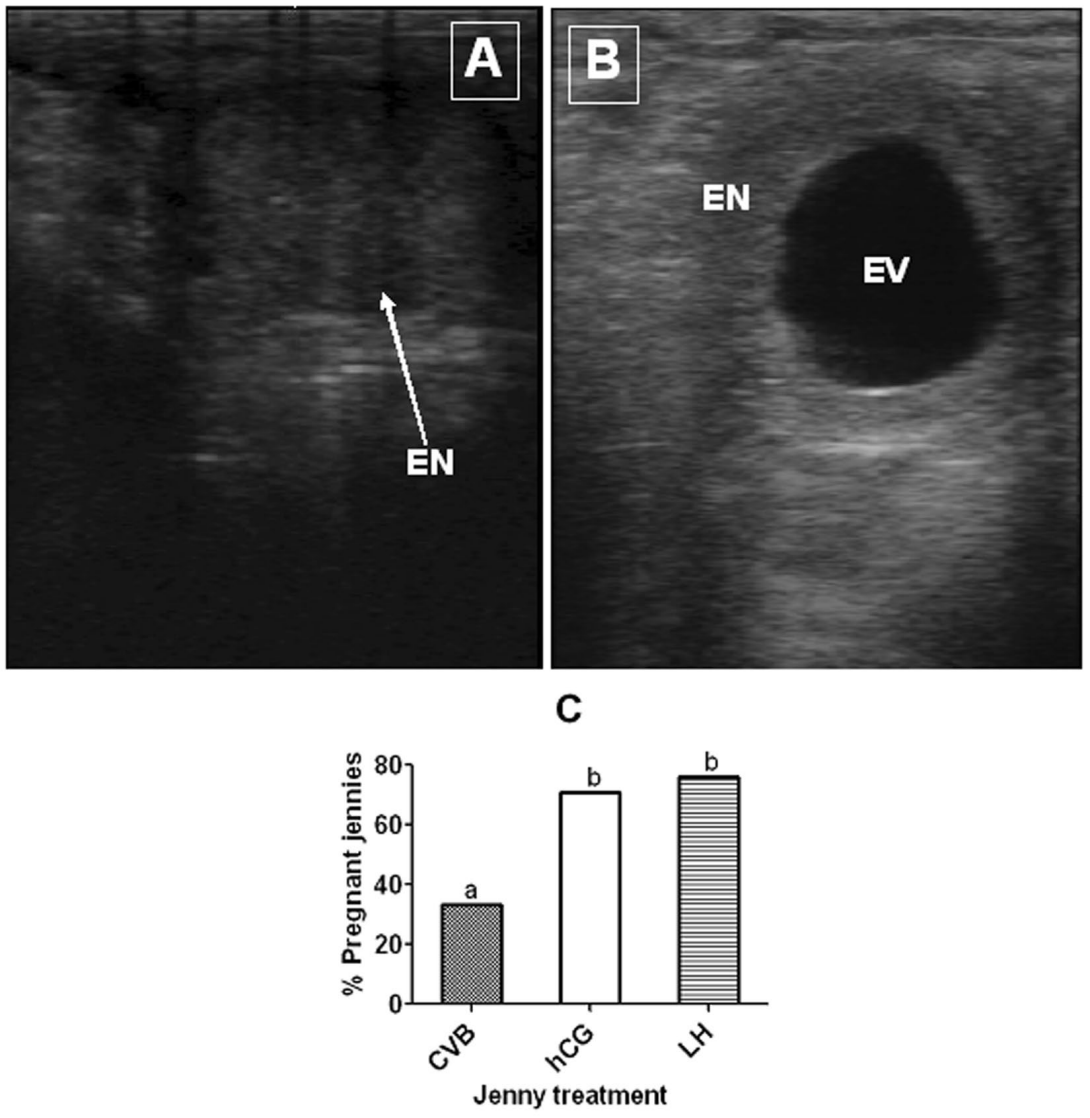
Conception rates of jennies following different treatments. To observe conception rates after insemination, LH- (n = 21) or hCG-treated (n = 24) jennies were artificially inseminated twice with frozen semen (10 straws of 5 × 10^7^ sperm) at 28 and 40 h after hormone injection. The conventionally bred (CVB) jennies (n = 33) were inseminated two or three times at 24-h intervals up to a rectal palpated ovulation. Conception was determined by ultrasonic imaging 15 to 20 days after insemination. (**A**) Ultrasonographic image of a non-pregnant uterine horn with endometrium (EN) obvious throughout the horn. (**B**) Ultrasonographic image of normal pregnant uterine horn observed on day 18 of gestation with apparent embryonic vesicle (EV) and endometrium (EN). (**C**) Conception rates of jennies following different treatments. Values with a different letter above bars differ significantly (P < 0.05).

To observe the effects of different ovulation induction treatments on conception rates, the LH- or hCG-treated jennies were artificially inseminated twice with frozen semen (10 straws of 5 × 10^7^ sperm) at 28 and 40 h after the hormone injection. Conception was determined by ultrasonic imaging 15 to 20 days after insemination. Ultrasonographic images of a non-pregnant uterine horn and a normal pregnant uterine horn are shown in Fig. 2A,B, respectively. Our observations (Fig. 2C) showed that conception rates after insemination were 76% in the LH-treated jennies and 70.8% in the hCG-treated jennies, which were significantly (P < 0.01) higher than that in the conventionally-bred jennies (33.3%).

### A comparison of FSH contents between the LH and hCG preparations used in this study

To answer the question why the LH preparation was better than the hCG preparation in synchronizing ovulations of jennies, contents of FSH, LH and hCG were compared by radioimmunoassay between the LH and hCG preparations used in this study. Three samples from the LH or hCG preparations were analyzed by radioimmunoassay for FSH, LH and hCG concentrations. The results showed that although the LH and hCG preparations contained predominantly LH and hCG, respectively, as expected, the LH preparation contained about 18 mIU/ml more FSH than the hCG preparation did (Table 1).

**Table 1:** Concentrations of FSH, LH and hCG contained in the LH and hCG preparations used for jenny treatment.

Preparations assayed	Hormone contents (mIU/ml)		
	FSH	LH	hCG
LH	18.43 ± 0.19^a^	160.81 ± 3.63^a^	0.41 ± 0.01^a^
hCG	0.35 ± 0.39^b^	0.33 ± 0.37^b^	>200^b^

a,b: Values with a different letter in superscripts differ significantly (P < 0.05).

### Effects of FSH supplementation and rainy days during ovulation induction treatment on distribution of ovulations

**Figure 3:**
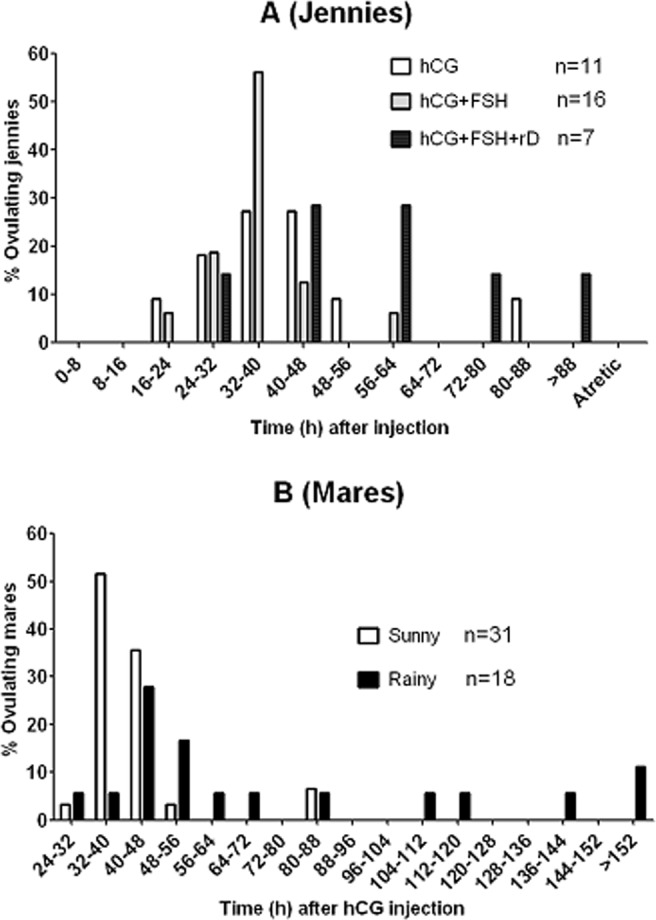
Effects of FSH supplementation and rainy days during ovulation induction treatment on distribution of ovulations in jennies or mares treated by hCG injection. In graph (**A**), when the dominant follicle diameter on an ovary reached 35 mm, jennies were injected intramuscularly with 5 ml saline containing 3000 IU hCG alone (the hCG group, n = 11) or with 50 IU FSH on sunny days (the hCG + FSH group, n = 16) or on rainy days (the hCG + FSH + rD group, n = 7), respectively. In graph (**B**), when the diameter of dominant follicle reached 35–40 mm, mares were injected intramuscularly with 3000 IU hCG on sunny days (n = 31) or on rainy days (n = 18). Follicle development was checked by ultrasonic imaging every 8 h after the injection until the dominant follicle was ovulated.

To study whether it was the FSH contained in the LH preparation that facilitated ovulation synchronization, jennies were treated with 3000 IU hCG alone or with 50 IU FSH on sunny days. The FSH dose of 50 IU was used because our calculation of the data in Table 1 indicated that 400 IU of LH contained about 40 IU of FSH. To examine ovulation distribution, follicle development was checked by ultrasonic imaging every 8 h after the injection until the dominant follicle was ovulated. The results demonstrated that of the jennies treated with hCG alone, 27.3% ovulated either from 32 to 40 h or 40 to 48 h, 18.2% ovulated from 24 to 32 h, and 9.1% ovulated either from 16 to 24 h, from 48 h to 56 h or from 80 to 88 h following the injection (Fig. 3). Among the jennies treated with hCG + FSH, 56.3% ovulated between 32 and 40 h, 18.8% ovulated between 24 and 32 h, 12.5% ovulated between 40 and 48 h, and 6.3% ovulated either between 16 and 24 h or between 56 and 64 h after injection. Thus, while about 73% of the jennies treated with hCG alone ovulated from 24 h to 48 h after injection, about 75% of the jennies treated with hCG plus FSH ovulated from 24 h to 40 h after the treatment, confirming that FSH supplementation had facilitated ovulation synchronization of jennies.

To observe the effects of the weather during the ovulation induction treatment on distribution of ovulations, some of the jennies were treated with hCG plus FSH on rainy days. The results showed that 28.6% of the jennies treated on rainy days ovulated from 40 to 48 h or from 56 to 64 h, and 14.3% ovulated either from 24 to 32 h, 72 to 80 h or after 88 h after the injection (Fig. 3A). Thus, compared to those in jennies treated with hCG plus FSH on sunny days, ovulations in jennies treated on rainy days were significantly postponed and scattered over a long period of time. Furthermore, our calculation of ovulation windows taking all the ovulations into account indicated that ovulations occurred within 72-h, 48-h and 72-h windows in the hCG, hCG + FSH and hCG + FSH + rainy day groups, respectively, confirming further that while FSH facilitated, rainy days impaired ovulation synchronization. Our experiments carried out from May 14 to June 20, 2017 in the Xinjiang region also indicated that while about 90% (28/31) of the mares treated on sunny days ovulated between 24 and 48 h after hCG injection, only 40% (7/18) of the mares treated on rainy days ovulated during the same period (Fig. 3B).

### Numbers of ovulated eggs following different treatments of jennies to induce ovulation

Our observations indicated that most of the jennies ovulated one egg following the ovulation induction treatments. Thus, only one of the 11 jennies in the control group and one of the 25 jennies in the LH group (shown in Fig. 1), and one of the 16 jennies in the hCG + FSH group (Fig. 3A) ovulated two eggs. Two of the 25 jennies in the hCG group (Fig. 1) ovulated two eggs. Thus, the average numbers of ovulated eggs after different treatments ranged from 1.04 ± 0.2 to 1.09 ± 0.3 (Mean ± SD).

## Discussion

Since the aim of the ovulation induction treatment is to accurately predict ovulation time, differences in ovulation synchronization among different treatments were emphasized in this study. The results showed that ovulation was better synchronized with LH or hCG plus FSH than with hCG alone. Thus, we observed that while 76% of the jennies in the hCG group ovulated between 24 and 48 h, 84% in the LH group ovulated between 24 and 40 h after injection. While 73% of the jennies treated with hCG alone ovulated from 24 h to 48 h after injection, 75% of the jennies treated with hCG plus FSH ovulated from 24 h to 40 h after the treatment. Furthermore, our calculation on the duration of ovulation windows, which took all the ovulations into account, showed that the ovulation window was narrower in the LH or hCG + FSH than in the hCG alone groups. In mares, Barbacini *et al*.^4^ observed more than 90% of ovulations within 48 h of treatment with hCG when a ≥35 mm follicle was detected, and 75% of the ovulations occurred between 25 and 48 h after treatment. In jennies, Carluccio *et al*.^13^ reported 92–100% of ovulations within 48 h of hCG treatment with 83–87% occurring between 25 and 48 h after hCG treatment.

The present results demonstrated that the LH preparation was more efficient than the hCG preparation in facilitating ovulation synchronization of jennies, and that the LH preparation contained about 18 mIU/ml more FSH than the hCG preparation did. Furthermore, FSH supplementation during the hCG treatment for ovulation induction facilitated ovulation synchronization. The results suggested that the high efficiency of the LH preparation compared to that of the hCG preparation was due to its FSH contents. Although the action of LH on ovulation induction has been well accepted, the potential role of FSH on ovulation induction has not been well established. In the mouse, LH promoted oocyte maturation by promoting production of EGF-like growth factors in granulosa cells^14,15^. The EGF-like growth factors act by binding the EGF receptor^16^. It was shown that FSH increased the number of EGF receptors and the binding of EGF on granulosa cells^17^. In mares, administration of hCG enhanced the expression of EGF-like growth factors amphiregulin and epiregulin^18^. Furthermore, FSH supplementation significantly increased the number of ovulations within 48 h after hCG treatment of mares showing 28 mm follicles^11^.

This study showed that ovulation induction treatment of jennies or mares during the rainy days significantly postponed the time and impaired synchronization of ovulations compared to that performed on sunny days. Despite great efforts, we found only one paper mentioning that a sudden bad weather would interrupt estrus and that cold weather would extend the estrous cycle in mares^19^. There are two potential mechanisms for rainy days to affect ovulation: humidity and photoperiod. It was reported that the humidity in an animal facility affected the embryo yield in laboratory animals^20^, but in large animals, it was the temperature and humidity index (THI) that affected ovulation. For example, Bilego *et al*.^21^ found that THI interfered with follicular and luteal dynamics and the response of heifers to the GnRH-PGF_2α_ treatment, leading to reduced pregnancy after insemination. Ghuman *et al*.^22^ reported that buffalo exhibited subestrus during low-breeding season at maximum ambient temperatures and relative humidity ranging from 36–45 °C and 30–80%, respectively. Because it was chiller on rainy days than on sunny days during our treatment, it was unlikely that our rainy days impaired ovulation through the THI pathway.

Another possibility for rainy days to impair ovulation of our jennies was that it might have affected the photoperiodicity. Photoperiod is an important factor that affects the annual sexual cycles of animals. For example, when two photoperiodic cycles were applied per year, ewes experienced two annual breeding seasons instead of one^23^. Exposure of ewes to as few as 70 days of photoperiodic information a year is sufficient to synchronize their circannual rhythm of reproductive neuroendocrine activity^24^. The mare is a long-day seasonal breeder with reproductive cyclicity occurring from early May to October in northern latitudes^23^. In the northern hemisphere, increasing the daylight ratio up to 16 h in November and December advances the beginning of the sexual season in the mare with first ovulation occurring up to 3 months earlier than under natural photoperiod. Such induced cycles are endocrinologically normal and fertile^25^.

This study showed that the conception rates were significantly higher in the LH- (76%) and hCG-treated jennies (70.8%) than in the conventionally bred jennies (33.3%). As for the reason for the high conception rates we achieved in jennies after the ovulation induction treatment, we attributed it to the high degree of ovulation synchronization that led to a high degree of synchronization between insemination and ovulation. Thus, 76% of the hCG-treated jennies and 84% of the LH-treated jennies would ovulate at times (24 h to 48 h after hormone injection) very close to times of insemination (28 h and 40 h after hormone injection). In the conventionally bred jennies, however, although the ovulation time was estimated by rectal palpation of preovulatory follicles, the accuracy was not guaranteed and might be affected by many factors such as skill differences between individuals. It was reported that mare conception rates were significantly affected by the insemination-ovulation interval. For example, while conception rates were 100% and 78.1%, respectively, when mares were inseminated 6–12 h or 12–24 h before ovulation, the conception rates decreased to 58.8% and 33.3%, respectively, when insemination took place 24–36 h or 48 h before ovulation^26^.

In summary, in searching for an alternative to hCG injection for ovulation induction in equids, we found that treatment of jennies with LH brought about a better ovulation synchronization than treatment with hCG and that ovulation induction with either LH or hCG followed by timed insemination significantly increased conception rates compared to those in conventionally bred jennies. Our further observation indicated that the LH preparation we used contained more FSH than the hCG preparation did, and supplementing the hCG treatment with FSH significantly improved ovulation synchronization. Furthermore, compared to those in jennies treated with hCG plus FSH on sunny days, ovulations in jennies treated the same way on rainy days were significantly postponed and less synchronized. It is thus concluded that jenny conception could be significantly improved by inducing ovulation with LH or hCG treatment followed by timed insemination and that FSH and the weather during treatment had profound effects on ovulation induction in jennies. The data are important not only for improving the efficacy of ovulation induction but also for understanding the mechanisms for follicular maturation and ovulation in equids.

## Materials and Methods

The experimental procedures were approved by the Animal Care and Use Committee of the Shandong Agricultural University P. R. China (Permit number: SDAUA-2001-0510). The methods were carried out in accordance with the approved guidelines. Unless otherwise specified, all chemicals and reagents used in the present study were purchased from Sigma Chemical Co. (St. Louis, MO, USA).

### Animals and detection of estrus and ovulation

All the experiments were carried out in Dong E E Jiao donkey institute. While experiments 1 and 2 were performed from April 5 to June 18 of 2014, experiments 3 and 4 were conducted from April 27 to May 27 of 2018. Dezhou jennies, between 3 and 10 years of age and in good body conditions, were kept in open paddocks and fed hay ad libitum. Signs of behavioral estrus were detected by daily teasing using a vigorous jack. Beginning at the first detection of estrus, ovarian activity and follicular growth were monitored by rectal ultrasonography (HS-2000, 7.5 MHz linear transducer, Honda Electronics Co., Ltd.) The monitoring was initially performed every 24 h (at 08:00), but changed to every 8 h (at 08:00, 16:00 and 24:00) after a follicle had developed to 35 mm in diameter, until ovulation. In this study, jennies were always treated on sunny days but some jennies were treated on rainy days in the experiments where the effect of rainy days was observed. Sunny and rainy days were defined as sunny or rainy for the first 48 h after hCG or LH injection.

### Hormone preparation and injection

The LH (Catalogue numbers: 130921), hCG (Catalogue numbers: 131023 and 180302), FSH (Catalogue number: 180325) and saline used in this study were all purchased from Hangzhou Animal Medicine Factory, Hangzhou, China. For injection, 400 IU of LH, 3000 IU of hCG or 3000 IU hCG plus 50 IU FSH were dissolved in 5 ml of saline. Intramuscular injections were conducted immediately after the detection of a 35-mm follicle. All the hormone injections were performed on sunny days except for those to test the effect of the weather on ovulation induction where injections were carried out on rainy days that lasted for at least 3 days.

## Artificial insemination

The jennies were inseminated with frozen-thawed semen by the conventional insemination technique into the uterine body, using a modified catheter. The semen dose for each insemination consisted of 10 of 0.5-ml straws containing 5 × 10^7^ spermatozoa. The sperm motility of the post-thawed semen was 35% to 40%. The LH- and hCG-treated jennies were inseminated twice at 28 and 40 h after the hormone injection. The conventionally bred jennies were inseminated at estrus when the dominant follicle had reached a preovulatory state as judged by rectal palpation and the insemination was conducted two or three times at 24-h intervals up to a rectal palpated ovulation. Conception was determined by ultrasonic imaging 15 to 20 days after insemination.

### Radioimmunoassay

Levels of LH, hCG and FSH in the LH and hCG preparations used in this study were measured by radioimmunoassay at the Central Hospital of Tai’an City using commercial kits. The LH, hCG and FSH concentrations were measured using an Iodine[^125^I]- Human Luteinizing Hormone Radioimmunoassay Kit from Union Medical & Pharmaceutical Technology Ltd., Tianjin, China, a Human chorionic gonadotropin-β (HCG-β) Radioimmunoassay kit from Beijing North Institute of Biological Technology, Beijing, China, and a Iodine[^125^I]- Human Follicle Stimulating Hormone Radioimmunoassay Kit from Union Medical & Pharmaceutical Technology Ltd., Tianjin, China, respectively. The intra- and inter-assay CVs for all kits were <10% and <15%, respectively. The limits of sensitivity for LH, hCG and FSH kits were 0 to 2000 mIU/ml, 4 to 160 mIU/ml and 0 to 200 mIU/ml, respectively.

### Data analysis

Data of hormone concentrations from radioimmunoassay were analyzed by one-way ANOVA, and were expressed as mean ± SD. The percentage data for conception rates were analyzed by chi-square test. The software used was SPSS 19 (SPSS Inc.). P < 0.05 was considered significant.

## References

[1] Voss JL (1975). The effect of HCG on duration of oestrus, ovulation time and fertility in mares. J Reprod Fertil Suppl..

[2] Michel TH, Rossdale PD, Cash RS (1986). Efficacy of human chorionic gonadotrophin and gonadotrophin releasing hormone for hastening ovulation in thoroughbred mares. Equine Vet J..

[3] Blanchar TL, Brinsko SP, Rigby SL (2002). Effects of deslorelin or hCG administration on reproductive performance in first postpartum estrus mares. Theriogenology.

[4] Barbacini S, Zavaglia G, Gulden P, Marchi V, Necchi D (2000). Retrospective study on the efficacy of hCG in an equine artificial insemination program using frozen semen. Equine Vet Edu..

[5] Sieme H, Schäfer T, Stout TA, Klug E, Waberski D (2003). The effects of different insemination regimes on fertility in mares. Theriogenology.

[6] Roser JF, Kiefer BL, Evans JW, Neely DP, Pacheco D (1979). A. The development of antibodies to human chorionic gonadotrophin following its repeated injection in the cyclic mare. J Reprod Fertil Suppl..

[7] Duchamp G, Bour B, Combarnous Y, Palmer E (1987). Alternative solutions to hCG induction of ovulation in the mare. J Reprod Fertil Suppl..

[8] Irvine DS, Downey BR, Parker WG, Sullivan JJ (1975). Duration of oestrus and time of ovulation in mares treated with synthetic Gn-RH (AY-24, 031). J Reprod Fertil Suppl..

[9] Oxender WD, Noden PA, Pratt MC (1977). Serum luteinizing hormone, estrus, and ovulation in mares following treatment with prostaglandin F2α and gonadotrophin-releasing hormone. Am J Vet Res..

[10] Becker SE, Johnson AL (1992). Effects of gonadotrophin-releasing hormone infused in a pulsatile or continuous fashion on serum gonadotropin concentrations and ovulation in the mare. J Anim Sci..

[11] Cox TJ, Squires EL, Carnevale EM (2009). Effect of follicle size and follicle-stimulating hormone on ovulation induction and embryo recovery in the mare. J Equine Vet Sci..

[12] Yen SS, Llerena O, Little B, Pearson OH (1968). Disappearance rates of endogenous luteinizing hormone and chorionic gonadotropin in man. J Clin Endocrinol Metab..

[13] Carluccio A (2007). Efficacy of hCG and GnRH for inducing ovulation in the jenny. Theriogenology.

[14] Park JY (2004). EGF-like growth factors as mediators of LH action in the ovulatory follicle. Science.

[15] Ashkenazi H (2005). Epidermal growth factor family members: endogenous mediators of the ovulatory response. Endocrinology.

[16] Johnson GR, Kannan B, Shoyab M, Stromberg K (1993). Amphiregulin induces tyrosine phosphorylation of the epidermal growth factor receptor and p185erbB2. Evidence that amphiregulin acts exclusively through the epidermal growth factor receptor at the surface of human epithelial cells. J Biol Chem..

[17] Prochazka R, Kalab P, Nagyova E (2003). Epidermal growth factor-receptor tyrosine kinase activity regulates expansion of porcine oocyte-cumulus cell complexes *in vitro*. Biol Reprod..

[18] Lindbloom SM, Farmerie TA, Clay CM, Seidel GE, Carnevale EM (2008). Potential involvement of EGF-like growth factors and phosphodiesterases in initiation of equine oocyte maturation. Anim Reprod Sci..

[19] Li XJ (1963). Characteristics and environmental influencing factors of estrus in mares (In Chinese). Chinese J Anim Sci..

[20] Diercks AK (2010). Environmental influences on the production of pre-implantation embryos. Theriogenology.

[21] Bilego UO (2013). Ovarian evaluation of Girolando (Holstein × Gir) heifers submitted to a GnRH-PGF2α-GnRH protocol in the dry or rainy seasons in the tropical savannah. Trop Anim Health Prod..

[22] Ghuman S, Honparkhe M, Singh J (2014). Comparison of ovsynch and progesterone-based protocol for induction of synchronized ovulation and conception rate in subestrous buffalo during low-breeding season. Iran J Vet Res..

[23] Hafez, E. S. E. Reproductive cycles. In: Hafez ESE. eds “*Reproduction in Farm Animals*”. 6^th^ Edition, Lea & Febiger, pp. 94–113 (1992).

[24] Woodfill CJI, Robinson JE, Malpaux B, Karsch FJ (1991). Synchronization of the circannual reproductive rhythm of the ewe by discrete photoperiodic signals. Biol Reprod..

[25] Oxender WD, Noden PA, Hafs HD (1977). Estrus, ovulation, and serum progesterone, estradiol, and LH concentrations in mares after an increased photoperiod during winter. Am J Vet Res..

[26] Cheng PL (1961). The application of some investigations of reproductive physiology in horse-breeding in China. Chin J Agri Sci..

